# Development of 3D Bioactive Scaffolds through 3D Printing Using Wollastonite–Gelatin Inks

**DOI:** 10.3390/polym12102420

**Published:** 2020-10-20

**Authors:** Filis Curti, Izabela-Cristina Stancu, Georgeta Voicu, Horia Iovu, Cristina-Ioana Dobrita, Lucian Toma Ciocan, Rodica Marinescu, Florin Iordache

**Affiliations:** 1 Advanced Polymer Materials Group, Faculty of Applied Chemistry and Material Science, University Politehnica of Bucharest, 1-7 Gh. Polizu Street, 011061 Bucharest, Romania; filis.curti@upb.ro (F.C.); izabela.stancu@upb.ro (I.-C.S.); horia.iovu@upb.ro (H.I.); 2 Department of Science and Engineering of Oxide Materials and Nanomaterials, Faculty of Applied Chemistry and Material Science, University Politehnica of Bucharest, 1-7 Gh. Polizu Street, 011061 Bucharest, Romania; 3 Department of Biomaterials and Medical Devices, Faculty of Medical Engineering, University Politehnica of Bucharest, 1-7 Gh. Polizu Street, 011061 Bucharest, Romania; ioana.dobrita@stud.fim.upb.ro; 4 Department of Prosthetics Technology and Dental Materials, “Carol Davila” University of Medicine and Pharmacy, 8 Eroii Sanitari Street, 050474 Bucharest, Romania; tehnologia-protezelor@umfcd.ro; 5 Department of Orthopedics, “Carol Davila” University of Medicine and Pharmacy, 8 Eroii Sanitari Street, 050474 Bucharest, Romania; rodicamarinescu@ymail.com; 6 Faculty of Veterinary Medicine, University of Agronomic Sciences and Veterinary Medicine of Bucharest, 105 Splaiul Independentei, 050097 Bucharest, Romania; flori.iordache@icbp.ro

**Keywords:** wollastonite, fish gelatin, paste-type ink, bioactive scaffold, 3D printing

## Abstract

The bioactivity of scaffolds represents a key property to facilitate the bone repair after orthopedic trauma. This study reports the development of biomimetic paste-type inks based on wollastonite (CS) and fish gelatin (FG) in a mass ratio similar to natural bone, as an appealing strategy to promote the mineralization during scaffold incubation in simulated body fluid (SBF). High-resolution 3D scaffolds were fabricated through 3D printing, and the homogeneous distribution of CS in the protein matrix was revealed by scanning electron microscopy/energy-dispersive X-ray diffraction analysis (SEM/EDX) micrographs. The bioactivity of the scaffold was suggested by an outstanding mineralization capacity revealed by the apatite layers deposited on the scaffold surface after immersion in SBF. The biocompatibility was demonstrated by cell proliferation established by MTT assay and fluorescence microscopy images and confirmed by SEM micrographs illustrating cell spreading. This work highlights the potential of the bicomponent inks to fabricate 3D bioactive scaffolds and predicts the osteogenic properties for bone regeneration applications.

## 1. Introduction

Three-dimensional (3D) printing has been developed as one of the most promising fabrication techniques able to produce patient-personalized scaffolds. The possibility to control porosity and interconnectivity provides a valuable tool to enhance cell-biomaterial or tissue-specific interactions, including the access and distribution of cells into the scaffold core, and to improve the transportation of nutrients and oxygen [1,2,3,4,5,6,7]. Currently, the main strategies in bone tissue engineering are focused on the development of 3D printed scaffolds based on biomimetic composites, with high precision and reproducibility [3,6,8,9,10,11]. The extracellular matrix (ECM) of bone is a natural composite consisting of hydroxyapatite (inorganic phase) and type I collagen (main organic component) [10,11,12]. The development of ceramic/polymer composites is a well known approach to imitate the natural bone tissue [9,11,12,13]. The organic component of such composites was often based on hydrogel materials since they replicate the structural and functional properties of native ECM [14,15]. Hydrogel precursors were widely used in ink formulations due to their printability and easy crosslinking, ensuring the stability and integrity of the printed structures [11,16]. Gelatin, a naturally derived biopolymer from collagen, has been extensively proposed due to its arginine–glycine–aspartic acid (RGD) sequence, biocompatibility, biodegradability, and variety from multiple sources [8,12,14,17,18]. While ink formulations are typically prepared with mammalian gelatins, religious issues or the risk of bovine spongiform encephalopathy transmitting may bring to attention the use of other types of gelatin [19,20,21]. Fish gelatin (FG) can be chosen as an interesting option for biomedical applications, as we previously reported for injectable solutions for electrospinning [19,20]. However, these protein components are not applicable on their own due to the insufficient mechanical properties. Therefore, the formulation of composite materials as biomimetic scaffolds for bone repair is an appealing solution to enhance the mechanical behavior [11,18]. An essential property for bone regeneration is the bioactivity of the scaffold. The incorporation of bioactive ceramic materials is expected to stimulate the osseointegration of the scaffolds due to the osteoconduction achieved by bone cell growth [1,11]. A wide range of bioactive ceramics, such as calcium phosphates, bioactive glass and calcium silicates, with similarities in composition to the inorganic phase of bone tissue were used to fabricate composite scaffolds for bone tissue engineering [1,4,5,22,23]. The main combination of biomaterials for composite scaffolds fabrication as bone tissue mimics was based on calcium phosphates as an inorganic component and hydrogels precursors as organic phase [3,12,17]. Hydroxyapatite, β-tricalcium phosphate or a combination of them, were broadly used to produce 3D printed composites due to their intrinsic bioactivity and osteoconductivity [2,3,6,8,9,10,12,17,22,24]. Apart from those, calcium silicates are another important bioceramic class which have gained considerable attention due to their outstanding bioactivity for biomimetic surface mineralization [22,23,24,25,26]. Wollastonite (CaO–SiO_2_, CS), one of the calcium silicates, can be considered a promising alternative for bone tissue repair due to its biocompatibility and bioactivity [22,24,27,28]. Compared with calcium phosphates, CS has higher bioactivity and great potential to induce osteoinduction and osseointegration [22,27]. By releasing Ca and Si ions, CS can induce a beneficial effect on cell growth, enhancing cell proliferation and differentiation [22,23,25,27]. Currently in the literature there are limited studies about the formulations of CS-based inks for the fabrication of 3D scaffolds, compared with the literature on calcium phosphates for 3D printing applications. For instance, promising 3D printed CS scaffolds were fabricated starting from a polyvinyl alcohol aqueous solution as a binder for the CS powder [7]. Inks based on sodium alginate, collagen type I and Mg-doped CS were prepared for osteochondral interface tissue engineering [22], while inks based on polyvinyl alcohol and strontium-containing Mg-doped CS, were used to produce 3D structures with applications in the bone tissue engineering field [27]. However, the formulation of CS-based inks is still poorly documented, and no significant data were reported for CS dispersion in polymeric precursors to induce bioactivity properties. This work reports the formulation of bicomponent 3D-printing precursors with a ratio of the organic/inorganic phase similar to that of bone [29]. The shape stability and fidelity are the main technical challenge in 3D printing and therefore thick paste-type injectable precursors based on CS powder dispersed in a concentrated aqueous FG solution were investigated. To the best of our knowledge, the formulation of wollastonite/fish gelatin (CS–FG) composite precursors to produce biomimetic 3D scaffolds with controllable architecture was not reported yet. As an approach for in vitro bioactivity assessment, the biomimetic surface mineralization correlated with the apatite formation on the scaffold surface was investigated by immersion in simulated body fluid (SBF). The fabricated scaffolds were characterized for their morpho-structural features through scanning electron microscopy (SEM) coupled with energy dispersive X-ray spectrometer (EDX). Preliminary in vitro tests were performed to determine the biocompatibility of CS–FG scaffolds.

## 2. Materials and Methods

### 2.1. Materials

FG (gelatin from cold water fish skin) and glutaraldehyde (50% aqueous solution, GA) were supplied by Sigma-Aldrich (St. Louis, MI, USA). CS powder was obtained by mixing calcium nitrate tetrahydrate (Ca(NO_3_)_2_⋅4H_2_O, ≥99.0%, Reag. ACS, Sigma-Aldrich) and tetraethyl orthosilicate (TEOS, C_8_H_20_O_4_Si, 99%, Reag. ACS, Sigma-Aldrich), at a CaO/SiO_2_ molar ratio of 1. SBF was prepared according to Kokubo’s proposed method [30,31]. All reagents were purchased from Sigma-Aldrich and were used as received. A GFL distiller apparatus was used to obtain double distilled water (ddw).

### 2.2. Methods

#### 2.2.1. CS Obtaining

CS powder was obtained by a sol–gel method described in a previously reported study [32]. Briefly, the method involved heating treatment at 1000 °C for 3 h and subsequently rapid cooling treatment at room temperature. The obtained material was grinded 30 min in a laboratory planetary ball mill (*v*_rot_ = 150 rot/min), resulting in a powder that was analyzed in terms of main crystalline phases and fineness. The crystalline phases were assessed by X-ray diffraction using a Shimadzu XRD 6000 diffractometer (with Ni-filtered Cu Kα radiation, λ = 1.5406 Å, with a scan step of 0.02°) and the fineness was assessed by laser granulometry using a Malvern Mastersizer 2000 laser granulometer.

#### 2.2.2. Ink Preparation

A 47 wt. % FG aqueous solution was prepared by gradual dissolution under stirring at 40 °C, followed by ultrasonication for 20 min. Bicomponent precursors were prepared by the stepwise incorporation of CS within the viscous FG solution, under mechanical continuous homogenization using a spatula. To obtain printable precursors containing around 40% organic matrix (similar to organic/mineral ratio in bone), with paste-type consistency and mechanical similarity with hard tissues, preliminary work was required to concentrate the FG solution as much as possible while still allowing the homogeneous incorporation of the CS phase. The formulations are described in Table 1.

#### 2.2.3. 3D Printing

The 3D structures based on CS–FG precursors were fabricated in a layer-by-layer assembly using the Direct Dispenser DD135N printhead from a multi-headed 3D Discovery Bioprinter (RegenHU, Villaz-Saint-Pierre, Switzerland). A five-layer rectangular object of 10 × 10 mm^2^ base was designed; the deposition direction of 0°–90° and the distance between the two adjacent filaments of 1 mm were set. The formulated precursors were loaded into syringes of 3 mL, and conical 25-gauge nozzles with a 0.25 mm inner diameter were attached. The scaffolds were printed on a glass microscope slide, at room temperature. The proposed inks were evaluated in terms of shape retention ability by varying and optimizing the printing parameters; the extrusion pressure ranged from 250 ± 30 kPa to 550 ± 30 kPa and the feed rate varied between 0.5 and 10 mm s^−1^.

The CS and FG printed structures were crosslinked to prevent protein dissolution and to provide stability in body fluids, by exposure to GA vapors for 21 days at room temperature.

#### 2.2.4. Morpho-Structural Characterization

Morphological and microstructural characterization of the CS powder and of the 3D printed scaffolds (precoated with gold) was performed using a FEI Quanta Inspect F50 scanning electron microscope (SEM, 1.2 nm resolution), coupled with energy-dispersive X-ray diffraction analysis (EDX).

#### 2.2.5. Mechanical Properties Evaluation

The mechanical properties were preliminarily investigated using a Brookfield CT3 texture analyzer with a 4500 g cell load (Brookfield Engineering) in compression mode. Hydrated crosslinked 3D printed scaffolds were used (their dimensions were measured using a digital caliper). The test was performed in triplicate. The samples were uniaxially compressed at a compression speed of 0.1 mm/s. The value of the compressive Young modulus was determined as the slope of the initial linear part of the compression curve, at a strain of 1.5%.

#### 2.2.6. Evaluation of the Mineralization Capacity

The in vitro mineralization capacity of the CS–FG3 printed scaffolds was investigated based on the methods described in the literature data [33,34,35]. Briefly, the scaffolds were immersed in individual test vials containing SBF at 37 °C, using a solid/liquid ratio of 0.5 mg mL^−1^ in the vials. After 7 and 14 days, respectively, the printed scaffolds were taken out and carefully washed to avoid the remaining soluble salts from the SBF. The results of the mineralization test were assessed morpho- and microstructurally by SEM using the method described in Section 2.2.4. The elemental mapping monitored the formation of a new mineral phase, while the ratio Ca/P was measured. In addition, Fourier transform infrared spectroscopy (FTIR) analysis was performed on the 3D printed scaffolds, before and after incubation in SBF for 7 and 14 days. Nano-hydroxyapatite, HAp, (Sigma Aldrich) was used as a control. A JASCO 4200 spectrometer equipped with a Specac Golden Gate attenuated total reflectance (ATR) device was used, and the spectra were registered in the wavenumber regions of 4000–600 cm^−1^ at a resolution of 4 cm^−1^.

#### 2.2.7. In Vitro Evaluation of Cell Behavior

##### MTT Assay for Cell Viability

Cell viability on CS–FG was assessed using human mesenchymal amniotic fluid stem cells (AFSCs). AFSCs were cultured in Dulbecco’s modified Eagle medium (DMEM) (Sigma-Aldrich) supplemented with 10% fetal bovine serum, 1% penicillin–streptomycin (Sigma-Aldrich), with the culture medium changed twice a week. The biocompatibility was assessed using an MTT reduction test (3-[4,5-dimethylthiazol-2-yl]-2,5-diphenyl tetrazolium bromide) (Vybrant^®^ MTT Cell Proliferation Assay Kit, Thermo Fischer Scientific, Waltham, MA, USA). Briefly, the AFSCs were seeded in 96-well tissue culture plates, using 3 × 10^3^ cells per well in the presence of CS–FG samples and incubated at 37 °C in humidified atmosphere with 5% CO_2_. Evaluation was performed at 24, 48 and 72 h post-seeding. At the end of the incubation period, MTT was added (10 µL solution 12 mM) and incubated 4 h at 37 °C. Then, the solubilization of formazan crystals was performed and after 1 h the absorbance at 570 nm was read using a TECAN Infinite M200 (Männedorf, Switzerland) microplate reader.

##### GSH-Glo Test for Toxicological Responses and Fluorescence Microscopy

A GSH-Glo Glutathione assay (GSH-GloTM, Promega) was used. AFSCs were seeded in 96-well plates, at a density of 3 × 10^3^ cells per well, in 300 µL DMEM supplemented with 10% fetal bovine serum and 1% penicillin, streptomycin/neomycin. After 24 h seeding, the cells are treated with samples and incubated for 72 h. Then, 100 µL 1X GSH-Glo reagent was added and incubated for 30 min at 37 °C, followed by the addition of 100 µL Luciferin detection reagent and incubation 15 min at 37 °C. After being well homogenized, the culture plate was read on a Microplate Luminometer Centro LB 960 (Berthold, Germany).

Fluorescence microscopy was also used to explore the biocompatibility of CS–FG scaffolds. The RED CMTPX fluorophore (Thermo Fischer Scientific, Waltham, MA, USA) tracker was added for the investigation of the viability and morphology of AFSCs seeded on CS–FG for one day. CMTPX fluorophore was added at a final concentration of 5 µM, incubated for 30 min, followed by AFSCs washing with PBS. Imaging was performed using a digital camera Olympus CKX 41 driven by CellSense Entry software (Olympus, Tokyo, Japan). All tests were performed in triplicate.

Additional morphological details describing the cells’ attachment and spreading on the surface of the 3D printed scaffolds were assessed by SEM investigations, using the equipment described in Section 2.2.4. Morpho-structural characterization.

Data were reported as the mean ± SD (standard deviation) or the mean ± SEM (standard error of mean), n indicating the number of samples. Statistical significance was assessed using either the two-tailed Student’s *t* test for the independent samples or its non-parametric variant (Mann–Whitney test), according to the results of the normality tests, for the quantitative data, and two-tailed Fisher’s exact probability test for the categorical frequency data. Statistical analysis was performed using OriginPro 7.5 software. For all the statistical tests, we set a critical level *p* < 0.05. All tests were performed in triplicate.

## 3. Results and Discussion

### 3.1. Characterization of CS Powder

**Figure 1:**
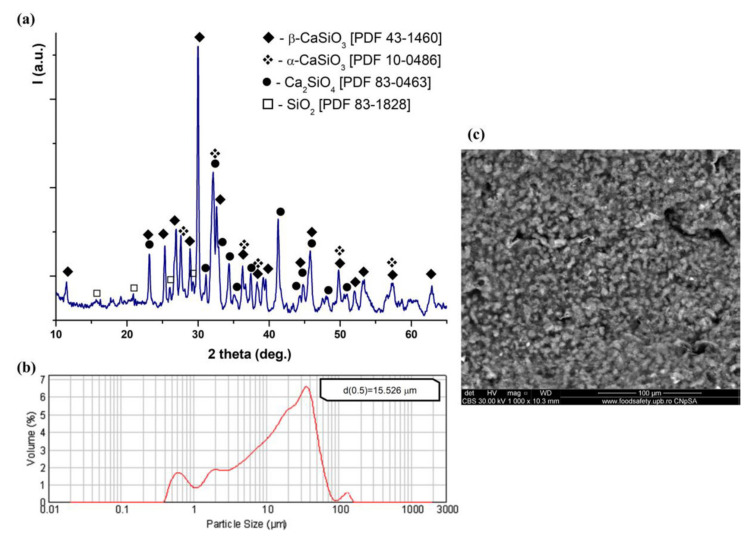
(**a**) The XRD pattern for the obtained CS powder; (**b**) the particle size distribution of CS powder; (**c**) the SEM micrograph representative for the CS phase (backscatter mode).

CS powder was obtained using a sol–gel method as previously described [32]. The main crystalline phases suggested by the XRD pattern (Figure 1a) of CS powder, have included wollastonite (CaSiO_3_ or CS; β-CS, PDF 43-1460; α-CS, PDF 10-0486), calcium orthosilicate (Ca_2_SiO_2_ or C_2_S, PDF 83-0463) and silicon oxide (SiO_2_, PDF 83-1828). The formation of CS can be described by the following reaction: C_2_S + SiO_2_ → 2CS. The largest quantity was represented by β-CS, which provides a better bioactivity due to its structure [36,37,38,39]. The average particle size of the obtained CS powder was approximatively 15.5 µm (Figure 1b). The morpho-structural appearance of the CS was that of a rather compact and agglomerated nanostructured granular phase, as presented in Figure 1c.

### 3.2. Fabrication of CS–FG Scaffolds

Maintaining shape fidelity and integrity are key challenges in the fabrication of 3D printed scaffolds. Therefore, CS–FG precursors (Table 1) were assessed through multiple printing trials. The lack of air bubbles in the ink-containing syringe and the ink homogeneity were checked as they are essential for scaffold reproducibility. The parameters’ optimization for each precursor was performed to determine the printable inks for a successful fabrication of the predesigned 3D structures. The evaluation of the precursors was indicated in Table 2, using “-” for the composition with no printability, specifying the tried parameters, and “+” for the printable precursor, indicating their established parameters.

**Figure 2:**
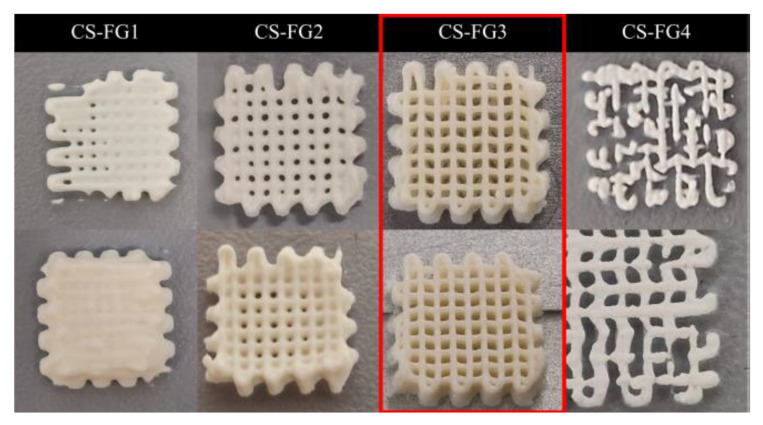
Digital images revealing the obtained structures during the 3D printing trials using the composite precursors based on FG and CS.

Although the extrusion pressure and the feed rate were adjusted many times, it was not possible to maintain the shape fidelity of the printed structures based on the CS–FG1 and CS–FG2 compositions, due to the strands collapse and filaments merging. In the case of the CS–FG4 precursor, the printed strands showed defects as they interrupted filaments, caused by the rapid drying of the extruded filament. However, the shape fidelity of the predesigned structure was obtained using the CS–FG3 composition, indicating that the mass ratio between the CS and FG was proper for the formulation of a printable ink. The printed objects using the CS–FG precursors were presented in Figure 2.

**Figure 3:**
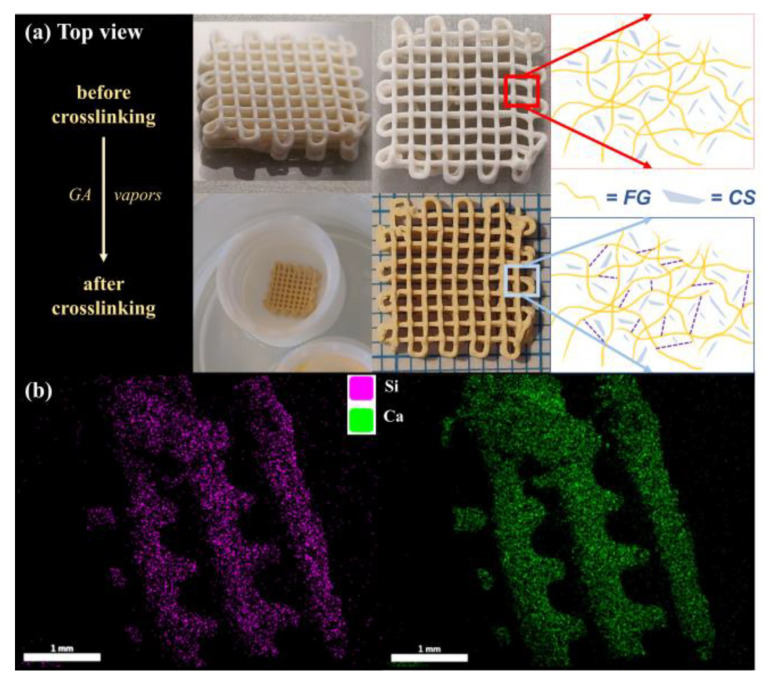
(**a**) Digital images revealing the top view of the CS–FG3 scaffolds and the schematic representation of the components, before and after the crosslinking process; (**b**) the SEM/EDX micrographs of the crosslinked CS–FG3 scaffold suggests a homogeneous distribution of CS in the gelatin matrix.

For subsequent analysis, the CS–FG3 ink was taken under consideration in the fabrication of the predesigned 3D scaffolds. The samples were submitted to a post-processing approach of crosslinking after printing, to prevent FG dissolution in aqueous fluids. A schematic representation of the crosslinking step was illustrated in Figure 3a, where the top view of uncrosslinked and crosslinked CS–FG3 samples was also presented.

### 3.3. Morpho-Structural Characterization

The distribution of the mineral phase in the CS–FG3 scaffolds was monitored through SEM/EDX analysis, as presented in Figure 3b. It can be noticed that the elemental mappings for Si and Ca elements were perfectly overlapping, highlighting that the CS component was homogenously dispersed in the protein matrix. This confirms the composite structure of the 3D-printed scaffold.

### 3.4. Mechanical Characterization

Following the uniaxial compression test, the compressive Young modulus was estimated to be 2.05 ± 0.34 MPa. This value indicates the efficiency of paste-like formulations in overcoming the mechanical weakness of simple gelatin hydrogels. For example, the compressive modulus of gelatin-based 3D printed scaffolds increased from 124 to 892 Pa when decreasing the strut spacing [40]. Another study reported a diminishing of the elastic modulus corresponding to the gelatin-based 3D printed scaffolds with about 90% compared to the bulk hydrogels (in the range of KPa for both types) [41].

### 3.5. Effect of CS–FG3 Scaffold on Bioactivity

**Figure 4:**
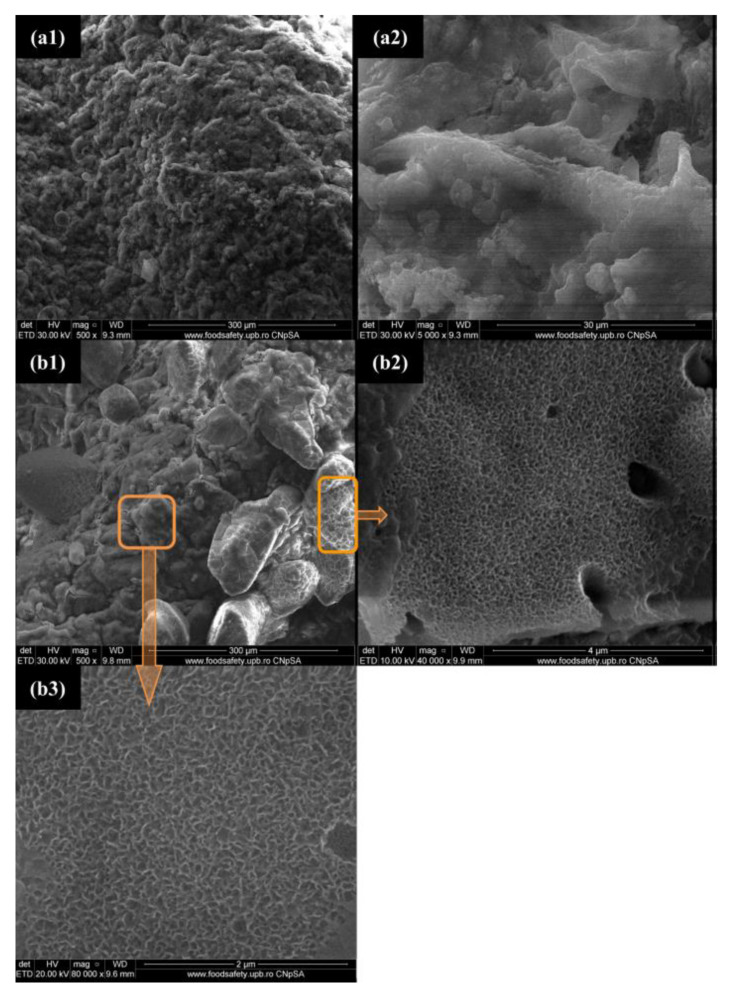
SEM micrographs of CS–FG3 scaffolds after 7 days (**a1**,**a2**) and 14 days (**b1**–**b3**) of immersion in simulated body fluid (SBF).

Due to the content of the bioactive CS, it was expected that the 3D printed scaffold would induce the formation of a bone-like mineral phase after incubation in SBF. From Figure 4, it can be noticed that continuous apatite-like layers with plate-like morphology were formed on the sample’s surfaces, following the immersion of CS–FG3 scaffolds in SBF from 7 to 14 days.

**Figure 5:**
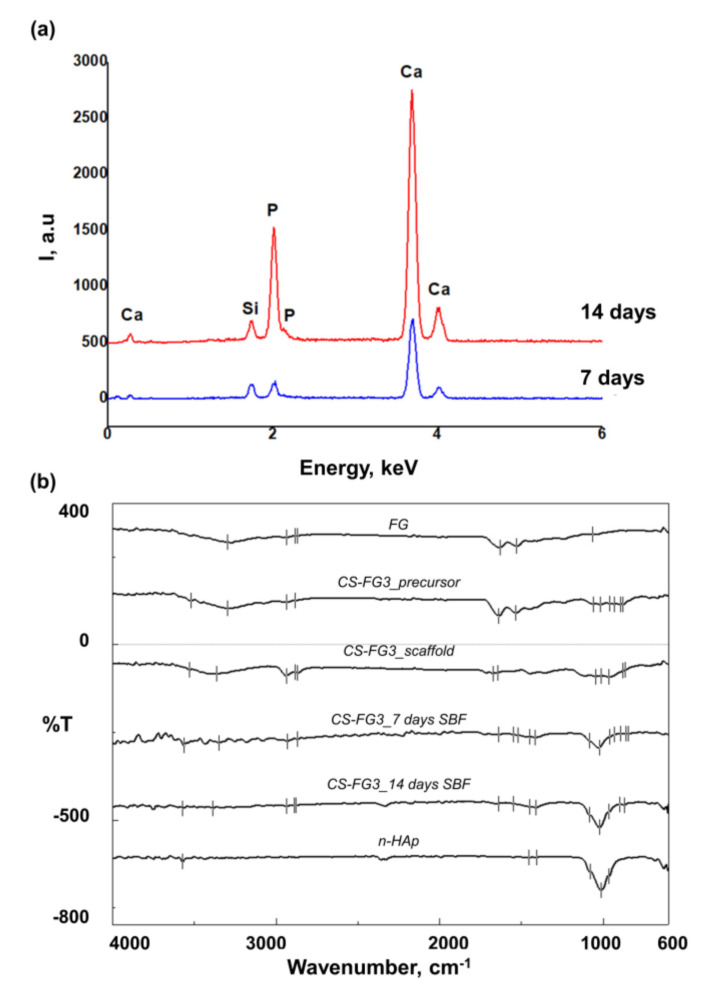
Representative structural information after the incubation of the samples in SBF: (**a**) EDX spectra; (**b**) FTIR–attenuated total reflectance (ATR) spectra recorded for the CS–FG3 scaffold before and after incubation for 7 and 14 days in SBF, compared to FG, the injectable precursor, and the control hydroxyapatite nanopowder.

FTIR spectra were recorded before and after the samples’ incubation in SBF for 7 and 14 days, respectively, as shown in Figure 5b. FG was used as a control for the protein phase, with its specific signals at 3297.68 cm^−1^ for amide A (N–H stretching vibration and -OH groups interacting through H bonding), 2937.06 and 2885.95 cm^−1^ assigned to C–H vibrations, 1631.48 and 1529.27 cm^−1^ assigned to amide I and amide II and an intense vibration of combined origin at 1063.55 cm^−1^. The spectrum of the precursor and that of the 3D printed scaffold before incubation in SBF presented combined signals for the two organic and inorganic phases. A broad peak centered at about 3530–3290 cm^−1^ was assigned to the combined O–H and N–H vibrations of the protein matrix, and C–H peaks were noticed at about 2940–2870 cm^−1^, with amide I and amide II at approximately 1680–1530 cm^−1^ (overlapping with water vibration), and a complex signal around 1060–1040 cm^−1^ similarly to other works [42]. The vibrations at 1017.27 and 962.305 cm^−1^ in the precursor and at 1012.45 and 963.269 cm^−1^ in the CS–FG3 scaffold correspond to Si–O; the peaks at 896.737 and 878.417 cm^−1^ in the precursor and at 878.417 and 864.917 cm^−1^ in the CS–FG3 scaffold are assigned to the stretching vibrations of Si–O–Si from the CS phase. These signals do not appear in the spectrum of gelatin; therefore, their presence was very easily noticed in the spectra of the injectable precursor and of the 3D printed CS–FG3 composite. Similar signals were reported by Shamsudin et al. for β-wollastonite [39]. After incubation in SBF, the protein signals (amide I, amide II) were reduced most probably due to the new surface-formed mineral phase. The SEM results combined with the FTIR spectral appearance suggest the formation of carbonated apatite. A weak signal centered at about 3570 cm^−1^ was noticed after the SBF interaction. It was previously reported [43] that the OH stretching of crystalline hydroxyapatite was weaker when compared to the high intensity of PO_4_ stretching vibrations typically centered at 1024 cm^−1^. Such peak distribution results from the apatite stoichiometry. The signals at about 1450–1410 cm^−1^ are specific to the high-energy vibrations of carbonate ions specific to carbonated hydroxyapatite. No major differences are visible between the spectra of the samples incubated for 7 and 14 days, only the signal assigned to the phosphate groups seem more intense at longer incubation in SBF, probably due to the thicker inorganic layer. Similar data were also reported for the bioactivity testing of β-wollastonite [39]. Furthermore, the spectra of HAp used as a control confirmed the apatite nature of the newly formed mineral phase.

**Figure 6:**
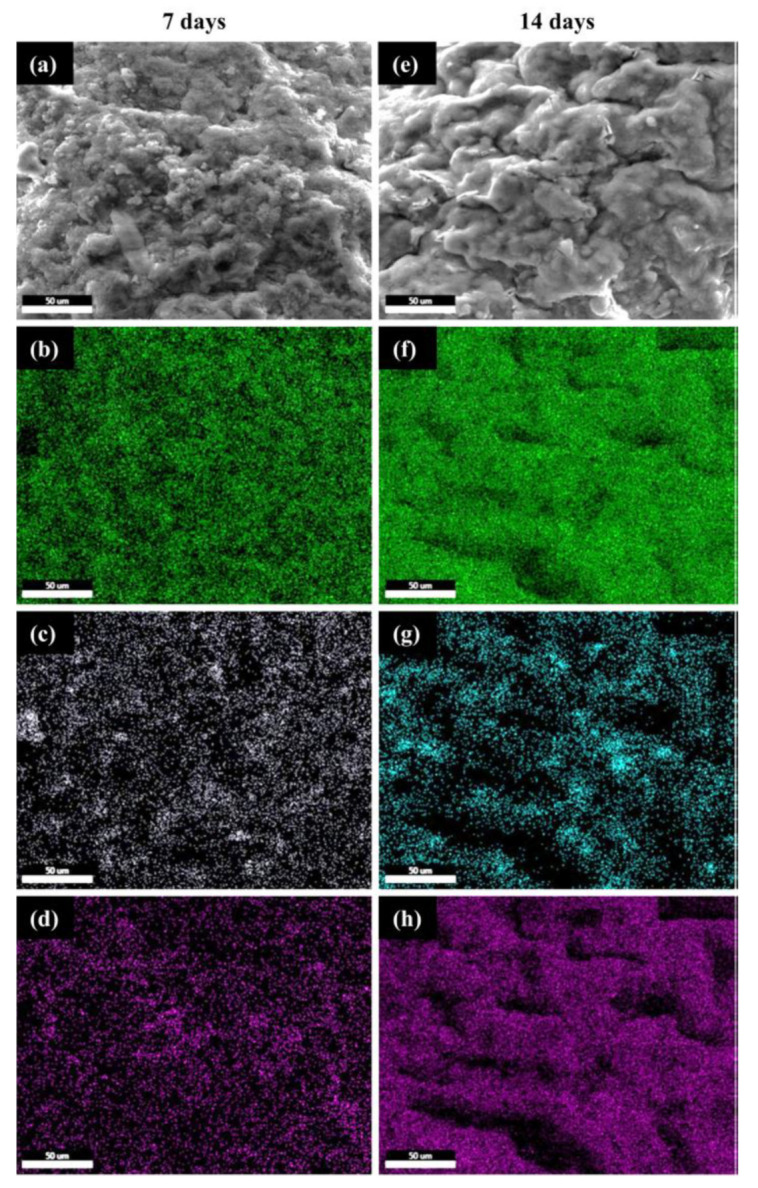
SEM/EDX micrographs with the elemental mapping for CS–FG3 scaffolds after 7 and 14 days of immersion in SBF (scalebar 50 μm): (**a**,**e**) SEM micrographs representative for the investigated areas, (**b**,**f**) Ca mapping, (**c**,**g**) Si mapping, and (**d**,**h**) P mapping.

Additional structural information regarding the apatite formation was provided by SEM/EDX micrographs, revealing the Si, Ca and P elements mapping at 7 and 14 days of immersion in SBF, as shown in Figure 6. The Ca/P ratio as determined by EDX (Figure 5a) was around 2.2, most probably due to the partial dissolution of the calcium from the CS phase when in contact with SBF. Typically, the CS will release calcium cations and silicate anions inducing an exchange with H^+^ and OH^−^ from the simulated fluid. The reactions and mechanism involved in the mineralization of β-wollastonite are explained in detail in [39].

The SEM/EDX elemental mapping suggested that after 7 days of immersion in SBF, the scaffold was homogeneously coated with a new mineral phase containing both Ca and P (common species from SBF), confirming the bioactivity of CS (Figure 6a–d). Furthermore, it is visible that after 14 days in SBF, the distribution of Si is less intense yet homogeneous, when compared to Ca and P perfectly overlapped higher-intensity maps. These evidences suggest that the mineralization process intensified from 7 to 14 days, as proved by the increase in the corresponding densities of Ca and P elements (Figure 6f,h). This is in good agreement with the previously discussed SEM and FTIR results.

### 3.6. Effect of CS–FG3 Scaffold on In Vitro Biocompatibility

**Figure 7:**
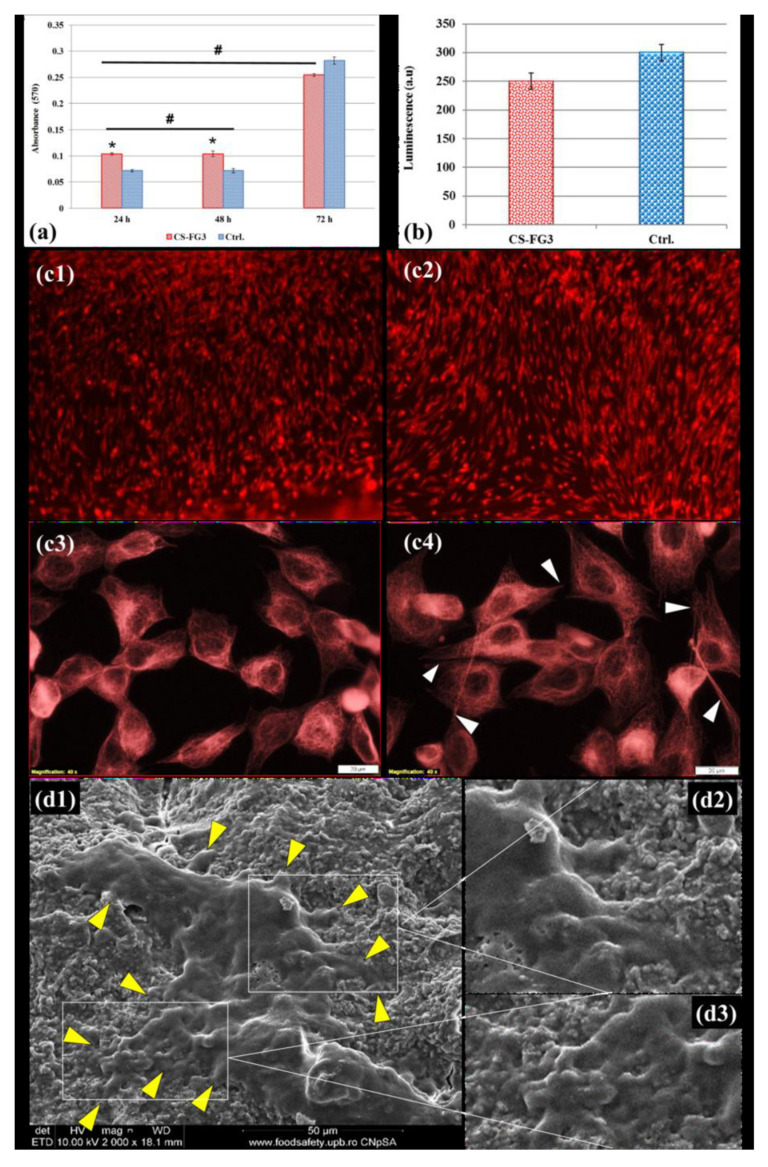
In vitro biocompatibility investigation: (**a**) cell viability results based on the MTT assay for the CS–FG3 scaffold and Ctrl (control culture) (results are represented as the mean ± standard error, n = 3, *p* < 0.05); (**b**) oxidative stress analysis for the Ctrl and CS–FG3 scaffold based on GSH-Glo assay (results are represented as the mean ± standard error, n = 3, *p* < 0.05); (**c**) fluorescence microscopy images at 72 h post-seeding, representative for cellular confluence (**c1**,**c2**) and for cellular morphological details (**c3**,**c4**): Ctrl (**c1**,**c3**), CS–FG3 scaffold (**c2**,**c4**); (**d1**) representative SEM micrograph of elongated well spread cells on the CS–FG3 scaffold, at 72 h post-seeding; arrow heads indicate cell attachment through filopodia; (**d2**,**d3**) morpho-structural details showing cellular filopodia on the FG–CS3 composite scaffolds.

In vitro biocompatibility tests were performed to investigate the influence of CS–FG3 printed scaffolds on cell adhesion. The cell viability results provided by the MTT test were shown in Figure 7a. In the case of CS–FG3 scaffolds, a significant cell proliferation occurred after 24 h and 48 h of incubation, compared to the control culture (Ctrl). It was also remarked that the cell division degree was no longer high after 48 h of incubation, and no major changes in cell viability were noticed between CS–FG3 and Ctrl.

The results of the MTT assay were confirmed by the fluorescence optical microscopy images that are shown in Figure 7c. After 24 h of incubation, it was clearly noticed that there was a much more important cell proliferation for CS–FG3 scaffolds in comparison with the Ctrl. The cells presented a normal morphology specific to the fibroblastic cell with an active phenotype. The extensions of fibroblast cells (visible in Figure 7(c4)) were possible due to the cytoskeleton activity, consisting mainly of actin filaments and microtubules. No dead cells or cell debris were revealed by the fluorescence optical microscopy images, highlighting that the CS–FG3 composite scaffold did not develop any cytotoxic effects. Moreover, the results of oxidative stress analysis illustrated in Figure 7b were lower in the case of the CS–FG3 scaffolds compared with Ctrl, after 72 h of incubation. The above results were also confirmed by SEM micrographs of the FG–CS3 scaffold after 72 h of cell incubation, as shown in Figure 7(d1–d3). The micrographs illustrated elongated cells, spread on the surface of the 3D printed scaffold, with well-developed filopodia anchored on the microstructured material. These results confirm the potential of the FG–CS3 scaffolds for tissue regeneration.

## 4. Conclusions

In this work, four precursors with different mass ratios between CS and FG were assessed in terms of shape retention ability for 3D printing. A biomimetic printable ink with the mass ratio of 77:23 (CS:FG) was proposed due to the shape fidelity of the printed scaffold, also mimicking the composition of the natural bone tissue. The CS–FG3 scaffold revealed a good compositional homogeneity, and promising bioactivity and biocompatibility features for bone tissue repair. The bioactivity of the CS–FG3 composite scaffold was proved by the newly deposited apatite layers on the scaffold’s surface, suggesting an outstanding mineralization capacity. The biocompatibility was confirmed through an important cell proliferation and a lower oxidative stress compared to the control culture, additionally evidenced by fluorescence optical microscopy and SEM micrographs. This work suggests the osteogenic potential of the 3D printed biomimetic CS–FG3 scaffold and its potential to stimulate bone regeneration. Further biological studies will deeper explore these aspects.

## Figures and Tables

**Table 1:** Three-dimensional printing precursors.

Code	CS:FG (wt:wt)
CS–FG1	68:32
CS–FG2	74:26
CS–FG3	77:23
CS–FG4	81:19

**Table 2:** Summary of the 3D printing trials.

Code	Needle Diameter (mm) = 0.25		
	Pressure (kPa)	Feed Rate (mm s^−1^)	Evaluation
CS–FG1	250–300	1–10	-
CS–FG2	300–350	1–10	-
CS–FG3	450–500	4–6	+
CS–FG4	550	0.5–3	-
